# A Secure and Lightweight Three-Factor-Based Authentication Scheme for Smart Healthcare Systems †

**DOI:** 10.3390/s20247136

**Published:** 2020-12-12

**Authors:** Jihyeon Ryu, Dongwoo Kang, Hakjun Lee, Hyoungshick Kim, Dongho Won

**Affiliations:** 1 Department of Software, Sungkyunkwan University, 2066 Seobu-ro, Jangan-gu, Suwon-si 16419, Gyeonggi-do, Korea; jhryu@security.re.kr; 2 Department of Electrical and Computer Engineering, Sungkyunkwan University, 2066 Seobu-ro, Jangan-gu, Suwon-si 16419, Gyeonggi-do, Korea; dwkang@security.re.kr (D.K.); hjlee@security.re.kr (H.L.); 3 Department of Computer Engineering, Sungkyunkwan University, 2066 Seobu-ro, Jangan-gu, Suwon-si 16419, Gyeonggi-do, Korea; hyoung@skku.edu

**Keywords:** authentication, WSN, healthcare, IoT

## Abstract

Internet of Things (IoT) technology has recently been integrated with various healthcare devices to monitor patients’ health status and share it with their healthcare practitioners. Since healthcare data often contain personal and sensitive information, healthcare systems must provide a secure user authentication scheme. Recently, Adavoudi-Jolfaei et al. and Sharma and Kalra proposed a lightweight protocol using hash function encryption only for user authentication on wireless sensor systems. In this paper, we found some weaknesses in target schemes. We propose a novel three-factor lightweight user authentication scheme that addresses these weaknesses and verifies the security of the proposed scheme using a formal verification tool called ProVerif. In addition, our proposed scheme outperforms other proposed symmetric encryption-based schemes or elliptic curve-based schemes.

## 1. Introduction

Digital healthcare services have recently received a considerable amount of attention as various Internet-enabled wearable devices have been deployed. Digital services can also be used to continuously monitor patients and share information with their healthcare practitioners. According to a Spyglass Consulting Group’s report [1], with 100 interviewees working in medical informatics and healthcare IT technology areas, about 88% of hospitals and healthcare systems have considered adopting remote patient monitoring (RPM) as their primary business model in the future.

RPM technology is increasingly used by hospitals and medical systems [1]. We believe that continuous monitoring and fast response times are necessary for high-risk patients with chronic diseases. For example, practitioners can use a monitoring device to collect ECG signals from patients with heart-related conditions and quickly identify any suspicious changes [2,3,4,5]. Because such data (e.g., raw ECG signals) are often highly personal and sensitive, any data that have been collected in healthcare systems should be securely protected and accessible only to authorized users such as primary care physicians. Furthermore, the scheme to monitor and analyze processes should not only be safe, but also be completed in real-time because patients might otherwise be at risk.

**Figure 1:**
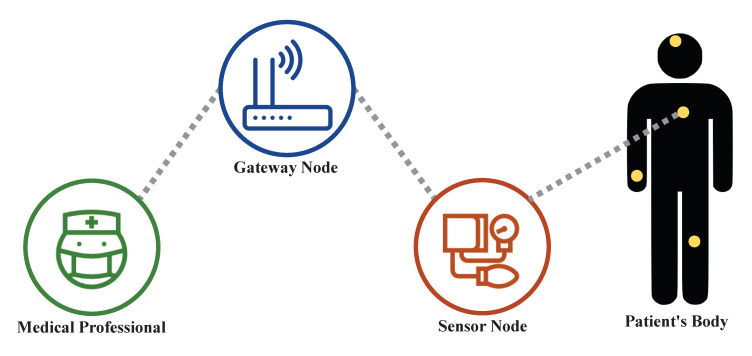
The Architecture of User Authentication for Digital Healthcare Services.

If there is a problem with the certification of these RPM technologies, the following damage can occur. First, if hospitals do not provide fast enough authentication using a heavy enough operation, quick feedback is impossible when monitoring the patient’s condition. In such cases, in a situation where urgent patients with fatal internal injuries require immediate treatment due to rapid changes, the treatment may be delayed due to the late certification speed. In the worst-case scenario, the treatment of patients can be difficult. Second, if there is a security flaw in the certification, privacy infringement of patients and medical personnel may occur. For example, if a session key is released, all medical information of the patient can be disclosed to the hacker. Therefore, in order to avoid such damage, a certification protocol is shown in Figure 1 that satisfies both high speed (i.e., lightweight computation) and safety should be used.

Many security protocols (e.g., [6,7,8,9]) have been developed to satisfy these security and performance requirements. Among those protocols, Sharma and Kalra’s scheme [6] is specifically designed to improve the protocol’s efficiency. Unlike existing protocols that require expensive cryptographic operations or three authentication factors [7,9,10], Sharma and Kalra’s scheme [6] uses hash functions with only two authentication factors. Similarly, Adavoudi-Jolfaei et al. [11] also proposed a protocol using hash functions only. Sharma and Kala’s scheme [6] and Adavoudi-Jolfei et al.’s scheme [11] are the most recently written lightweight authentication protocols that can keep high speed and safety, the conditions required by the healthcare system.

In this paper, we confirmed that Adavoudi-Jolfaei et al.’s scheme [11] has a severe vulnerability. We also demonstrate that Sharma and Kalra’s scheme [6] has a serious design error. Therefore, we propose a new scheme to fix the weaknesses of these target schemes. We formally verify the security of the new protocol using ProVerif, an automatic cryptographic protocol verifier. In summary, this paper presents the following contribution:

- We demonstrate that Sharma and Kalra’s scheme [6] has a serious design error: mutual authentication between a practitioner and a sensor cannot be ensured in their original protocol.
- We confirm that Adavoudi-Jolfaei et al.’s scheme [11] and Sharma and Kalra’s scheme have a severe vulnerability. We find that Adavoudi-Jolfaei et al.’s scheme [11] is vulnerable to user impersonation attack and session key attack. We also find that Sharma and Kalra’s scheme [6] is vulnerable to password guessing attack, stealing the session key and sensor impersonation attack.
- We propose a scheme for smart healthcare systems. Our new scheme resists privileged insider attack, outsider attack, offline ID guessing attack, online id guessing attack, session key disclosure attack, practitioner impersonation attack, and sensor impersonation attack. We provide security proofs.
- We formally verify the security of the new protocol using ProVerif, an automatic cryptographic protocol verifier.
- We show the performance analysis of the proposed scheme. We compared the proposed scheme with that of Chen et al. [12], Renuka et al. [13], and Li et al. [14] to show how efficient our proposed scheme is.

The remainder of this paper is arranged as follows: Section 2 describes related work. Section 3 introduces the preliminary knowledge necessary to understand the scheme by Sharma and Kalra and Adavoudi-Jolfaei et al. The target schemes are briefly described in Section 4. Section 5 discusses several weaknesses of the target schemes. We propose our improved scheme in Section 6 and show the security analysis of the proposed scheme in Section 7. In Section 8, we show the performance analysis of the proposed scheme. Finally, Section 9 concludes the paper.

## 2. Related Work

Various user authentication schemes have been proposed for smart healthcare applications.

Hu et al. [15] proposed a real-time hardware and software-based healthcare monitoring system for cardiac patients in 2007. The proposed scheme focuses on efficiency improvements but lacks adequate security protection. Malasri et al. [16] proposed an authentication scheme for wireless mote-based medical sensor networks using an ECC (elliptic curve cryptography) system in 2009. However, the scheme cannot withstand denial-of-service and relay nodes attacks. Furthermore, ECC may be too expensive for embedded devices in the medical domain.

In 2012, Kumar et al. [17] proposed a two-factor user authentication scheme for wireless medical sensors to monitor patients’ health status. However, Khan and Kumari [18] found that Kumar et al.’s scheme is vulnerable to security attacks. The scheme included the use of a smart card to enhance the security of the protocol, but the user information stored on the smart card can end up leaked if the smart card is stolen. Khan and Kumari proposed an improved scheme to fix the security flaws of the previous scheme in 2014. Li et al. [19] and Wu et al. [20] each analyzed the scheme presented by Khan and Kumari [18]. They discovered that Khan and Kumari’s scheme is not secure against offline password guessing attacks, as it does not identify invalid input and user impersonation attacks. Li et al. [19] and Wu et al. [20] proposed their respective improved schemes, which employ a smart card to overcome the security flaws of Khan and Kumari [18]. Hossain et al. [21] proposed an IoT-based ECG health monitoring service framework in the cloud. They presented a framework for secure transmission of patients data from different sensors to the cloud in a wireless environment.

Recently, Sharma and Kalra [6] proposed an authentication scheme for cloud-IoT-based remote patient healthcare monitoring services. It is efficient because only the hash function is used for system encryption. Several papers briefly addressed security flaws of Sharma and Karla’s scheme [22,23]. However, they only mentioned briefly that privileged insider attacks are possible. However, we have pinpointed the structural problems of Sharma and Kalra’s scheme, and showed that password guessing attack, stealing the session key attack, and sensor impersonation attack are possible in the case of privileged insider attack.

In 2016, Gope et al. [24] proposed a novel two-factor lightweight anonymous authentication protocol in WSNs (wireless sensor networks) that uses a database to overcome prior vulnerabilities. However, Adavoudi-Jolfaei et al. [11] argue that protocol is vulnerable to side-channel attacks because of the use of 2-factors, and that the session keys are also vulnerable. To overcome these drawbacks, in 2019, Adavoudi-Jolfaei et al. [11]. proposed a new 3-factor authentication protocol in WSN. Unfortunately, Shin and Kwon found user collusion attacks, desynchronization attack and no sensor node anonymity. In addition, through our prior research, we found more weaknesses that user impersonation attack and session key attack are able to take advantage of.

## 3. Preliminaries

This section introduces the hash function, fuzzy extractor and threat model used in this paper.

### 3.1. Hash Function

Data convert an arbitrary value to a fixed-length value through a hash function. This is useful for fast and safe search functions. The hash function has the following properties [25].

- Preimage-resistance It is computationally impossible to use the output of any hash value to find the input that results in this value, i.e., to find any preimage $a^{{}^{\prime }}$ such that $h(a^{{}^{\prime }})=b$ when given any *b* for which a corresponding input is not known.
- 2nd-preimage-resistance For any input, when there is an output for the hash function, it is computationally impossible to find another input value with this output, i.e., to find a 2nd-preimage $a^{{}^{\prime }}\neq a$ such that $h\left(a\right)=h\left(a^{{}^{\prime }}\right)$.
- Collision resistance It is computationally infeasible to find two different inputs with the same hashing result, i.e., any two distinct inputs *a*, $a^{{}^{\prime }}$, which hash to the same output, such that $h\left(a\right)=h\left(a^{{}^{\prime }}\right)$.

### 3.2. Fuzzy Extractor

Biometric information should be treated as sensitive. Since biometric information is unique to the user, it is convenient to use, but difficult to handle. In general, biometrics cannot be recognized equally each time. Therefore, a fuzzy extractor is used to recognize varied biometric information within a certain tolerance range. The fuzzy extractor can obtain a unique string using error tolerance. The fuzzy extractor operates through two procedures (Gen, Rep), as follows [26,27]:

(1) $$Gen\;\left(B\right)\to \langle \alpha ,\beta \rangle$$

(2) $$Rep\;(B^{*},\beta )=\alpha$$

Gen and Rep are a probabilistic generation function and a deterministic reproduction function, respectively. Gen returns a factored out string $\alpha \in {\left\{0,1\right\}}^{k}$ for the input biometrics *B* and a co-adjutant string $\beta \in {\left\{0,1\right\}}^{*}$. Rep is a function that restores β to α, and any vector $B^{*}$ close to *B*.

### 3.3. Threat Model

Based on the work of Dolev and Yao [28] and other previous research [10,29], we employ a threat model with the following assumptions.

- An attacker can steal a smart device with the user’s identity.
- An attacker can eavesdrop on a public channel. An attacker can steal the message between the user and the gateway node or between the gateway node and the sensor node.
- An attacker can extract the information stored in the smart device as a side-channel attack.

## 4. Review of Target Protocols

This section describes the target protocols.

### 4.1. Review of Adavoudi-Jolfaei et al.’s Protocol

This section describes the protocol developed by Adavoudi-Jolfaei et al. [11]. The scheme consists of four phases: registration, login, authentication, and password change. The notation for the target paper [11] is shown in Table 1.

#### 4.1.1. Registration Phase

In the registration phase, *U* and GWN in the private channel exchange secret information about SC. When the user authenticates, this allows confidential information to be stored in the database used by SC and GWN.

1. User *U* chooses his/her identity $U_{id}$ and sends the registration request $U_{id}$ and personal credential to the gateway node GWN in the secure channel.
2. The gateway node GWN generates a random number $n_{g}$, a unique random number used to identify a particular access group $G_{j}$, a random number user access privilege mask $APM_{j}$ and random sequence number $Ts_{ug}$. Then, the created variables are grouped as $G=\{G_{1}$, $G_{2}$, …}, $APM=\{APM_{1}$, $APM_{2}$, …}. After obtaining the registration request from user *U*, GWN calculates as follows:
  $\;\;\;\;Sk_{ug}=h$ ($U_{id}\|n_{g}$)$⊕GW_{id}$
  $\;\;\;\;sid_{j}=h$($U_{id}\left\|r_{j}\right\|Sk_{ug}$)
  $\;\;\;\;SU_{id}=\{sid_{1}$, $sid_{2}$, …}
  $\;\;\;\;KEM_{ug_{j}}=h$ ($U_{id}\left\|sid_{j}\right\|r_{j}^{\prime }$)
  $\;\;\;\;G=\{G_{1}$, $G_{2}$, …}
  $\;\;\;\;APM=\{APM_{1}$, $APM_{2}$, …}
  $\;\;\;\;U_{id}^{\#}=U_{id}⊕h$ ($GWN_{id}\left\|w\right\|Ts_{ug}$)
  $\;\;\;\;Sk_{ug}^{\#}=Sk_{ug}⊕h$ ($GWN_{id}\left\|U_{id}\right\|w$)
  $\;\;\;\;G_{j}^{\#}=G_{j}⊕h$ ($GWN_{id}\left\|U_{id}\right\|w$)
  $\;\;\;\;APM_{j}^{\#}=APM_{j}⊕h$ ($GWN_{id}\left\|U_{id}\right\|w$)
  $\;\;\;\;Sk_{gs}^{\#}=Sk_{gs}⊕h$ ($GWN_{id}\left\|w\right\|SN_{id}$)
  $\;\;\;\;KEM_{ug}^{\#}=KEM_{ug}⊕h$ ($GWN_{id}\left\|U_{id}\right\|w$) using its secret key *w*.
  The data are saved $\langle Ts_{ug},$ ($SU_{id}$, $KEM_{ug}^{\#}$), $Sk_{ug}^{\#}$, $Sk_{gs}^{\#}$, $U_{id}^{\#}$, $G^{\#}$, $APM^{\#}\rangle$ in DB.GWN sends $\langle Sk_{ug}$, ($SU_{id}$, $KEM_{ug}$), $Ts_{ug}$, $G_{U}$, *h* (·)〉 to user *U* in SC.
3. After user *U* takes SC from the GWN, chooses his/her $U_{id}$, password $U_{psw}$, imprints the biometric $U_{b}$ and then computes as follows:
  $\;\;\;\;Gen\left(U_{b}\right)=(RS_{U}$, $P_{U})$
  $\;\;\;\;Sk_{ug}^{*}=Sk_{ug}⊕h$(h$(U_{id})⊕h$ ($U_{psw})⊕h$ $(RS_{U}))$
  $\;\;\;\;KEM_{ug}^{*}=KEM_{ug}⊕h$ (*h* ($U_{id})⊕h$ ($U_{psw})⊕h$ ($RS_{U}$))
  $\;\;\;\;SU_{id}^{*}=SU_{id}⊕h$ (*h* $(U_{id})⊕h$ $(U_{psw})⊕h$ $\left(RS_{U}\right)$)
  $\;\;\;\;G^{*}=G⊕h$ (*h* $(U_{id})⊕h$ $(U_{psw})⊕h$ $(RS_{U})$), $f_{U}^{*}=h$ (*h* $(Sk_{ug})⊕h$ $(U_{id})⊕h$ $(U_{psw})⊕h$ $(RS_{U})$)
  Moreover, save the data $\langle Sk_{ug}^{*}$, $f_{U}^{*}$, ($SU_{id}^{*}$, $KEM_{ug}^{*}$), Tsug, $G^{*}$, $P_{U}$, Gen (·), Rep (·), *h* (·)〉 in SC.

#### 4.1.2. Login Phase

In the login phase, the user enters his/her confidential information into the smart card and requests login.

1. *U* inserts the smart card and enters $U_{id}$, $U_{psw}$ and $U_{b}$. The smart card computes $RS_{U}=Rep$ ($U_{b}$, $P_{U})$, $Sk_{ug}=Sk_{ug}^{*}⊕h$ (h $(U_{id})⊕h$ $(U_{psw})⊕h$ $\left(RS_{U})\right)$ and checks the condition $f_{U}=h$ (h $(Sk_{ug})⊕h$ $(U_{psw})⊕h$ $(U_{id})⊕h$ $\left(RS_{U}\right))\overset{?}{=}f_{U}^{*}$. If it holds, the smart card ensures that the user successfully passes the verification process. Otherwise, this phase terminates immediately.
2. After successful verification, user *U* generates random number $N_{u}$ and the system computes as follows:
  $\;\;\;\;N_{x}=Sk_{ug}⊕N_{u}$
  $\;\;\;\;G=G^{*}⊕h$ (*h* ($U_{id})⊕h$ ($U_{psw})⊕h$ ($RS_{U}))$
  $\;\;\;\;AU_{id}=h$ ($U_{id}\|Sk_{ug}\|N_{u}\left\|Ts_{ug}\right)$
  $\;\;\;\;G_{j}^{{}^{\prime }}=G_{j}⊕N_{u}$
  $\;\;\;\;V_{1}=h$$\left(AU_{id}\|G_{j}^{{}^{\prime }}\|Sk_{ug}\|N_{x}\|SN_{id}\right)$
  If there is a loss of synchronization, user *U* selects one of the unused pair of ($sid_{j}^{*}$, $KEM_{{ug}_{j}}^{*}$) from ($SU_{id}^{*}$, $KEM_{ug}^{*}$) and surrenders his/her $U_{id}$, $U_{psw}$, $RS_{U}$ and computes $sid_{j}=sid_{j}^{*}⊕h$ (h $(U_{id})⊕h$ $(U_{psw})⊕h$ $\left(RS_{U})\right)$, $KEM_{ug}=KEM_{ug}^{*}⊕h$ (h $(U_{id})⊕h$ $(U_{psw})⊕h$ $\left(RS_{U})\right)$, $AU_{id}=sid_{j}$ and $Sk_{ug}=KEM_{{ug}_{j}}$.
3. *U* sends the login request messages $M_{A_{1}}=\{AU_{id}$, $G_{j}^{{}^{\prime }}$, $N_{x}$, $Ts_{ug}\left(ifreq\right)$, $SN_{id}$, $V_{1}\}$ to GWN.

#### 4.1.3. Authentication Phase

In the authentication phase, GWN verifies *U* with the login message received from *U*, and sends a new message containing secret information to SN. SN and *U* share their keys and exchange the secret information.

1. After receiving the login request messages $M_{A_{1}}$ from user *U*, GWN first checks the validity of the transaction sequence number $Ts_{ug}$. GWN computes as follows:
  $\;\;\;\;N_{u}=Sk_{ug}⊕N_{x}$
  $\;\;\;\;G_{j}=G_{j}^{{}^{\prime }}⊕N_{u}$
  h ($GWN_{id}\|U_{id}\left\|w\right)=G_{j}^{\#}⊕G_{j}$
  $\;\;\;\;APM_{j}=APM_{j}^{\#}⊕h$ ($GWN_{id}\left\|U_{id}\right\|w$) that $G_{j}^{\#}$ and $APM_{j}^{\#}$ are in DB.
  Then, GWN calculates $AU_{id}=h$ $\left(U_{id}\|Sk_{ug}\|N_{U}\|Ts_{ug}\right)$, $V_{1}=h$ $\left(AU_{id}\|G_{j}^{{}^{\prime }}\|Sk_{ug}\|N_{x}\|SN_{id}\right)$ and checks if $AU_{id}$ and $V_{1}$ are valid. If the verification of $AU_{id}$ is successful, then calculation continues. Otherwise, GWN terminates the session. GWN generates a session key SK and time stamp *T* and calculates as follows:
  $\;\;\;\;SK^{{}^{\prime }}=h$$(Sk_{gs})⊕SK$, $APM_{j}^{{}^{\prime }}=h$$Sk_{gs}⊕APM_{j}$ and
  $\;\;\;\;V_{2}=h$$\left(AU_{id}\|APM_{j}^{{}^{\prime }}\|SK^{{}^{\prime }}\|T\|Sk_{gs}\right)$
  Finally, GWN sends the messages $M_{A_{2}}=\{AU_{id}$, $APM_{j}^{{}^{\prime }}$, $SK^{{}^{\prime }}$, *T*, $V_{2}\}$ to the sensor node SN.
2. Upon receiving the message $M_{A_{2}}$, SN assess the validity of *T*. If it is not valid, SN disconnects the session. If it is valid, SN also verifies $V_{2}\overset{?}{=}h$ $\left(AU_{id}\|APM_{j}^{{}^{\prime }}\|SK^{{}^{\prime }}\|T\|Sk_{gs}\right)$. If this condition is not satisfied, SN disconnects the session. If it is satisfied, SN computes as follows:
  $\;\;\;\;APM_{j}=APM_{j}^{{}^{\prime }}⊕h$$\left(Sk_{gs}\right)$ and generates a new time stamp $T^{{}^{\prime }}$.
  SK=h$\left(Sk_{gs}\right)⊕SK^{{}^{\prime }}$, $V_{3}=h$$\left(SK\|Sk_{gs}\|SN_{id}\|T^{{}^{\prime }}\right)$
  $\;\;\;\;\;K_{{gs}_{new}}=h$$\left(Sk_{gs}\|SN_{id}\right)$ and
  $\;\;\;\;Sk_{gs}=K_{{gs}_{new}}$
  Finally, SN transmits $M_{A_{3}}=\{T^{{}^{\prime }}$, $SN_{id}$, $V_{3}\}$ to GWN.
3. The gateway node GWN checks that the time stamp $T^{\prime }$ and $V_{3}\overset{?}{=}h$ $\left(SK\|Sk_{gs}\|SN_{id}\|T^{{}^{\prime }}\right)$. If not, it terminates the connection. GWN generates a random number $Ts_{{ug}_{new}}$ and calculates as follows:
  Ts=h$(Sk_{ug}\|U_{id}\|N_{U}$)
  $\;\;\;\;SK^{\prime \prime }=h$$(Sk_{ug}\|U_{id}\|N_{U})⊕SK$
  $\;\;\;\;V_{4}=h$$\left(SK^{\prime \prime }\|N_{U}\|Ts\|Sk_{ug}\right)$
  $\;\;\;\;K_{{ug}_{new}}=h$$\left(Sk_{ug}\|U_{id}\|Ts_{{ug}_{new}}\right)$
  $\;\;\;\;Sk_{ug}=K_{{ug}_{new}}$
  $\;\;\;\;K_{{gs}_{new}}=h($$Sk_{gs}\left\|SN_{id}\right)$
  GWN updates $Sk_{ug}=K_{{ug}_{new}}$ and $Sk_{gs}=K_{{gs}_{new}}$. If GWN cannot find $Ts_{ug}$ in $M_{A_{1}}$, GWN generates a random number $K_{{ug}_{new}}$ and calculates x=h $\left(U_{id}\|KEM_{{ug}_{j}}\right)⊕K_{{ug}_{new}}$. Then, GWN updates $Sk_{ug}=K_{{ug}_{new}}$ and then sends the messages $M_{A_{4}}=\{SK^{\prime \prime }$, Ts, $V_{4}$, x} to the user *U*.
4. When user *U* obtains the message $V_{4}=h$ $\left(SK^{\prime \prime }\|N_{U}\|Ts\|Sk_{ug}\right)$, the protocol checks its validity. If there is no abnormality, the system proceeds to the next step or ends the session. Furthermore, *U* computes SK=h $Sk_{ug}\|U_{id}\left\|N_{U}\right)⊕SK^{\prime \prime }$, $Ts_{{ug}_{new}}=h$ $(Sk_{ug}\|U_{id}\|N_{U})⊕Ts$, $K_{{ug}_{new}}=h$ $(Sk_{ug}\|U_{id}\|Ts_{{ug}_{new}}$ and then updates $Sk_{ug}=K_{{ug}_{new}}$ and $Ts_{ug}=Ts_{{ug}_{new}}$.
5. *U* and SN have successfully shared SK. SN responds to user *U*’s query according to $APM_{j}$ stored for user *U* using session key SK. Finally, at the end of this phase, SN removes $APM_{j}$ from storage for security reasons.

#### 4.1.4. Password and Biometrics Change Phase

The protocol uses the following steps to change the user’s password:

1. *U* puts his/her smart card into the terminal and inserts $U_{id}$, previous password $U_{psw}$ and previous biometric $U_{b}$. *U* then inputs the new password $U_{psw}^{*}$ and new biometric $U_{b}^{*}$.
2. The smart card computes $RS_{U}=Rep$ $(U_{b}$, $P_{U})$ and retrieves $Sk_{ug}$, $KEM_{ug}$, $SU_{id}$, *G* and $f_{U}$. The smart card continues to compute as follows:
  $\;\;\;\;Sk_{ug}=Sk_{ug}^{*}⊕h$(h$(U_{id})⊕h$$(U_{psw})⊕h$$\left(RS_{U})\right)$
  $\;\;\;\;KM_{ug}=KEM_{ug}^{*}⊕h$(h$(U_{id})⊕h$$(U_{psw})⊕h$$\left(RS_{U})\right)$
  $\;\;\;\;SU_{id}=SU_{id}^{*}⊕h$(h$(U_{id})⊕h$$(U_{psw})⊕h$$\left(RS_{U})\right)$
  $\;\;\;\;G=G^{*}⊕h$(h$(U_{id})⊕h$$(U_{psw})⊕h$$\left(RS_{U})\right)$
  $\;\;\;\;f_{U}=f_{U}^{*}⊕h$(h$(Sk_{ug})⊕h$$(U_{psw})⊕h$$(U_{id})⊕h$$\left(RS_{U})\right)$
3. The smart card computes Gen $\left(U_{b}^{*}\right)$, $Sk_{ug}^{**}$, $SU_{id}^{**}$, $KEM_{ug}^{**}$, $G^{**}$ and $f_{U}^{**}$, as shown below.
  Gen$(U_{b}^{*})=$$(RS_{U}^{*}$, $P_{U}^{*})$
  $\;\;\;\;Sk_{ug}^{**}=Sk_{ug}⊕h$(h$(U_{id})⊕h$$(U_{psw}^{*})⊕h$$\left(RS_{U}^{*})\right)$
  $\;\;\;\;SU_{id}^{**}=SU_{id}⊕h$(h$(U_{id})⊕h$$(U_{psw}^{*})⊕h$$\left(RS_{U}^{*})\right)$
  $\;\;\;\;KEM_{ug}^{**}=KEM_{ug}⊕h$(h$(U_{id})⊕h$$(U_{psw}^{*})⊕h$$\left(RS_{U}^{*})\right)$
  $\;\;\;\;G^{**}=G⊕h$(h$(U_{id})⊕h$$(U_{psw}^{*})⊕h$$\left(RS_{U}^{*})\right)$
  $\;\;\;\;f_{U}^{**}=h$(h$(Sk_{ug})⊕h$$(U_{psw}^{*})⊕h$$(U_{id})⊕h$$\left(RS_{U}^{*})\right)$
4. Finally, the smart card replaces $Sk_{ug}^{*}$ with $Sk_{ug}^{**}$, $SU_{id}^{*}$ with $SU_{id}^{**}$, $KEM_{ug}^{*}$ with $KEM_{ug}^{**}$, $G^{*}$ with $G^{**}$, $f_{U}^{*}$ with $f_{U}^{**}$ and $P_{U}$ with $P_{U}^{*}$.

### 4.2. Review of Sharma and Kalra’s scheme

This section briefly describes Sharma and Kalra’s scheme. The notation of the scheme is summarized in Table 1. Sharma and Kalra’s scheme consists of five different phases:

1. Setup Phase: The registration center sets up the parameters.
2. Registration Phase: The practitioner registers with his/her identity and password.
3. Login Phase: The practitioner logs in with his/her identity, password and smart device.
4. Authentication Phase: The practitioner and sensor node mutually authenticate.
5. Password Change Phase: The practitioner inputs identity, password and smart device, and changes his/her old password to the new password.

#### 4.2.1. Setup Phase

The gateway node GWN obtains its secret key *K* from the registration center. The center also computes and gives $Sk_{gs}=h\left(SN_{id}\|K\right)$ to the sensor node SN. $Sk_{gs}$ and *K* are stored in GWN and SN.

#### 4.2.2. Registration Phase

The practitioner creates his/her identity and password. He/she registers through the gateway node to receive a smart device. The detailed process is as follows:

1. Practitioner *P* chooses his/her $ID_{p}$ and $PW_{p}$ and generates random number *R*, computes the masked password $Mask\left(PW_{p}\right)=h\left(PW_{p}\|R\right)$. Finally, he/she sends the registration message $\{Mask\left(PW_{p}\right)$, $ID_{p}\}$ to the gateway node GWN.
2. After the gateway node GWN receives the message from the practitioner, it computes variables $a=h(Mask\left(PW_{p}\right)\|ID_{p})$, $b=h(ID_{p}\|K)$, $c=h\left(K\right)⊕h(Mask\left(PW_{p}\right)\|b)$ and $d=b⊕h(Mask\left(PW_{p}\right)\|a)$. After calculation, GWN sends the smart device SC={a, *c*, d} to *P*.
3. *P* stores {a, *c*, *d*, R} in SC.

#### 4.2.3. Login Phase

When the practitioner enters his/her identity and password, the smart device checks that the practitioner is an authorized party. The procedure for doing so is as follows:

1. *P* inputs his/her identity $ID_{p}$ and password $PW_{p}$ in his/her smart device.
2. SC computes $Mask\left(PW_{p}\right)=h\left(PW_{p}\|R\right)$, $a^{{}^{\prime }}=h\left(Mask\left(PW_{p}\right)\|ID_{p}\right)$ and compares *a* to $a^{{}^{\prime }}$. If the two are not the same, *P* fails to login.

#### 4.2.4. Authentication Phase

We describe the mutual authentication of the practitioner’s login information and the sensor node. The procedure is as follows:

1. If *P* successfully logs in, SC computes $b=d⊕h(Mask\left(PW_{p}\right)\|a)$, $h\left(K\right)=c⊕h(Mask\left(PW_{p}\right)\|b)$, $V_{1}=ID_{p}⊕h\left(h\left(K\right)\|T_{1}\right)$. SC selects a random nonce *N*, and calculates $V_{2}=N⊕h\left(b\|T_{1}\right)$, $V_{3}=h(V_{1}\|V_{2}\left\|N\right\|T_{1})$. Finally, SC posts the message $M_{1}=\{V_{1}$, $V_{2}$, $V_{3}$, $T_{1}$, $SN_{id}\}$ to GWN.
2. GWN checks that the timestamp $|T_{1}-T_{c}|<\Delta T$. ΔT means maximum transmission delay. If it is in range, GWN chooses a random nonce *M* and computes:
  $\;\;\;\;MSN_{id}=SN_{id}⊕h\left(h\left(K\right)\|T_{2}\right)$
  $\;\;\;\;V_{4}=h(Sk_{gs}\|T_{1}\left\|T_{2}\right)⊕M$
  $\;\;\;\;V_{5}=h(SN_{id}\|V_{4}\|T_{1}\|T_{2}\left\|M\right)$
  Finally, GWN sends the message $M_{2}=\{V_{1}$, $V_{2}$, $V_{3}$, $V_{4}$, $V_{5}$, $T_{1}$, $T_{2}$, $MSN_{id}\}$ to the sensor node SN.
3. SN checks the validity of $|T_{2}-T_{c}|<\Delta T$. If it is valid, SN continues as follows:
  $\;\;\;\;MSN_{id}^{{}^{\prime }}=MSN_{id}⊕h\left(h\left(K\right)\|T_{2}\right)$, $Sk_{gs}^{{}^{\prime }}=h\left(SN_{id}\right)$
  $\;\;\;\;M^{{}^{\prime }}=V_{4}⊕h(Sk_{gs}^{{}^{\prime }}\|T_{1}\left\|T_{2}\right)$
  $\;\;\;\;V_{5}^{{}^{\prime }}=h(SN_{id}\|V_{4}\|T_{1}\|T_{2}\left\|M^{{}^{\prime }}\right)$
  $\;\;\;\;ID_{p}^{{}^{\prime }}=V_{1}⊕h\left(h\left(K\right)\|T_{1}\right)$, $b^{{}^{\prime }}=h\left(ID_{p}^{{}^{\prime }}\|K\right)$
  $\;\;\;\;N^{{}^{\prime }}=V_{2}⊕h\left(b^{{}^{\prime }}\|T_{1}\right)$
  $\;\;\;\;V_{3}^{{}^{\prime }}=h(V_{1}\|V_{2}\|N^{{}^{\prime }}\left\|T_{1}\right)$, $V_{6}=M^{{}^{\prime }}⊕h\left(b^{{}^{\prime }}\|T_{3}\right)$
  $\;\;\;\;V_{7}=N^{{}^{\prime }}⊕h\left(Sk_{gs}^{{}^{\prime }}\|T_{3}\right)$
  $\;\;\;\;V_{8}=h(V_{6}\|b^{{}^{\prime }}\left\|T_{3}\right)$
  $\;\;\;\;V_{9}=h(V_{7}\|Sk_{gs}^{{}^{\prime }}\left\|T_{3}\right)$
  Finally, SN posts the message $M_{3}=\{V_{6}$, $V_{7}$, $V_{8}$, $V_{9}$, $T_{3}\}$ to GWN.
4. GWN checks the timestamp $|T_{3}-T_{c}|<\Delta T$, and if it is valid, computes as follows:
  $\;\;\;\;V_{9}^{{}^{\prime }}=h(V_{7}\|Sk_{gs}\left\|T_{3}\right)$
  $\;\;\;\;N^{{}^{\prime }}=V_{7}⊕h\left(Sk_{gs}\|T_{3}\right)$
  $\;\;\;\;SK_{GWN}=h\left(N^{{}^{\prime }}⊕M\right)$
  $\;\;\;\;V_{10}=h(SK_{GWN}\|V_{6}\|V_{8}\|T_{3}\left\|T_{4}\right)$
  At the end of the computation, GWN sends the message $M_{4}=\{V_{6}$, $V_{8}$, $V_{10}$, $T_{3}$, $T_{4}\}$ to *P*.
5. *P* checks the timestamp $|T_{4}-T_{c}|<\Delta T$. If it is in range, *P* computes $V_{8}^{{}^{\prime }}=h(V_{6}\left\|b\right\|T_{3})$. It also computes $M^{{}^{\prime }}=V_{6}⊕h\left(b\|T_{3}\right)$, $SK_{p}=h\left(N⊕M^{{}^{\prime }}\right)$ and $V_{10}^{{}^{\prime }}=h(SK_{p}\|V_{6}\|V_{8}\|T_{3}\left\|T_{4}\right)$.

#### 4.2.5. Password Change Phase

The practitioner should be able to change his/her password if he/she wants to do so (e.g., for security reasons or because of a lost password). The procedure is as follows:

1. *P* inputs his/her $ID_{p}$ and $PW_{p}$ to SC.
2. SC computes $Mask\left(PW_{p}\right)=h\left(PW_{p}\|R\right)$, $a^{*}=h\left(Mask\left(PW_{p}\right)\|ID_{p}\right)$. SC verifies that $a^{*}=a$: if so, it computes $b=d⊕h(Mask\left(PW_{p}\right)\|a)$, $h\left(K\right)=h(Mask\left(PW_{p}\right)\|b)⊕c$. Finally, SC sends the message {Enternewpassword} to *P*.
3. *P* inputs his/her new password $PW_{p}^{new}$ to SC.
4. SC computes $Mask{\left(PW_{p}\right)}^{{}^{\prime }}=h\left(R\|PW_{p}^{new}\right)$, $a^{{}^{\prime }}=h\left(Mask{\left(PW_{p}\right)}^{{}^{\prime }}\|ID_{p}\right)$, $d^{{}^{\prime }}=b⊕h\left(Mask{\left(PW_{p}\right)}^{{}^{\prime }}\|a^{{}^{\prime }}\right)$, $c^{{}^{\prime }}=h\left(K\right)⊕h\left(Mask{\left(PW_{p}\right)}^{{}^{\prime }}\|b^{{}^{\prime }}\right)$. Finally, SC replaces {a, *c*, d} with $\{a^{{}^{\prime }}$, $c^{{}^{\prime }}$, $d^{{}^{\prime }}\}$.

## 5. Analysis of Target Schemes

### 5.1. Analysis of Adavoudi-Jolfaei et al.’s Scheme

In this section, we prove that the scheme put forth by Adavoudi-Jolfaei et al. [11] has some security vulnerabilities. The details are as follow.

#### 5.1.1. Loss of Smart Card Information

Attacker A can easily decrypt the information on the SC in the following two cases. The first case is an insider attack in the registration phase, while the second case is loss of synchronization in the login phase. Insider attack is the stronger of the two: it should be considered when there is no apparent loss of synchronization.

##### Insider Attack

In the registration phase, Attacker A extracts the smart card SC when GWN sends information to *U*. He/she can then read the information stored on the SC $\{Sk_{ug}$, $SU_{id}$, $KEM_{ug}$, $Ts_{ug}$, *G*, *h* (·)} that is not encrypted.

##### Loss of Synchronization

1. An attacker A steals *U*’s smart card SC, which contains sensitive information $\langle Sk_{ug}^{*}$, $f_{u}^{*}$, ($SU_{id}^{*}$, $KEM_{ug}^{*}$), Tsug, $G^{*}$, $P_{U}$, Gen (·), Rep (·), *h* (·)〉.
2. In the loss of synchronization case, A can thus see the user’s login message $M_{A_{1}}=\{AU_{id}$, $G_{j}^{{}^{\prime }}$, $N_{x}$, $Ts_{ug}\left(ifreq\right)$, $SN_{id}$, $V_{1}\}$. A computes as follows:
h (h $(U_{id})⊕h$ $(U_{psw})⊕h$ $\left(RS_{U}\right))=AU_{id}⊕SU_{id}^{*}$
$\;\;\;\;Sk_{ug}=Sk_{ug}^{*}⊕h$ (h $(U_{id})⊕h$ $(U_{psw})⊕h$ $\left(RS_{U})\right)$
$\;\;\;\;KEM_{ug}=KEM_{ug}^{*}⊕h$ (h $(U_{id})⊕h$ $(U_{psw})⊕h$ $\left(RS_{U})\right)$
$\;\;\;\;G=G^{*}⊕h$ (h $(U_{id})⊕h$ $(U_{psw})⊕h$ $\left(RS_{U})\right)$

#### 5.1.2. User Impersonation Attack

Attacker A can carry out a user impersonation attack (the victim is assumed to be *U*). The details are as follows.

1. A generates random numbers $N_{A}$ and computes:
  $\;\;\;\;N_{xA}=Sk_{ug}⊕N_{A}$
  $\;\;\;\;G_{jA}^{{}^{\prime }}=G_{j}⊕N_{A}$
  $\;\;\;\;AID_{A}=h$$\left(U_{id}\|Sk_{ug}\|N_{A}\|Ts_{ug}\right)$
  $\;\;\;\;V_{1A}=h$$\left(AU_{id}\|G_{jA}\|Sk_{ug}\|N_{A}\|SN_{id}\right)$
  $N_{xA}$, $G_{jA}^{{}^{\prime }}$, $AID_{A}$ and $V_{1A}$ from $Sk_{ug}$ and $G_{j}$ obtained from the stolen smart card attack.
2. A transmits the login request $M_{A1}=\{AID_{A}$, $G_{jA}^{{}^{\prime }}$, $N_{xA}$, $Ts_{ug}$, $SN_{id}$, $V_{1A}\}$ to the gateway node GWN.
3. After GWN obtains the login request from A, first, it verifies $Ts_{ug}$ and calculates:
  $\;\;\;\;N_{A}=Sk_{ug}⊕N_{xA}$
  $\;\;\;\;G_{j}=G_{jA}^{{}^{\prime }}⊕N_{A}$
  h ($GWN_{id}\|U_{id}\left\|w\right)=G_{j}^{\#}⊕G_{j}$
  $\;\;\;\;APM_{j}=APM_{j}^{\#}⊕h$ ($GWN_{id}\left\|U_{id}\right\|w$)
  $\;\;\;\;AID_{A}=h$$\left(U_{id}\|Sk_{ug}\|N_{A}\|Ts_{ug}\right)$
  $\;\;\;\;V_{1A}=h$$\left(AU_{id}\|G_{jA}^{{}^{\prime }}\|Sk_{ug}\|N_{A}\|SN_{id}\right)$
  GWN checks if $AID_{A}$ and $V_{1}$ is valid. GWN does not detect the presence of the attacker. Unfortunately, GWN still believes it is in communication with *U*.

As a result, attacker A will be verified as GWN by user *U*. Therefore, the user impersonation attack is successful.

#### 5.1.3. Session Key Attack

Assume that Attacker A has access to the DB. At this time, Attacker A can extract the session key SK of user *U* and sensor node SN as follows.

1. Assume that attacker A can access the database $DB=\langle Ts_{ug},$ ($SU_{id}$, $KEM_{ug}^{\#}$), $Sk_{ug}^{\#}$, $Sk_{gs}^{\#}$, $U_{id}^{\#}$, $G^{\#}$, $APM^{\#}\rangle$. He/she will use the data $Sk_{ug}^{\#}$.
2. Attacker A extracts the message $M_{A_{2}}=\{AU_{id}$, $APM_{j}^{{}^{\prime }}$, $SK^{{}^{\prime }}$, *T*, $V_{2}\}$ and calculates:
  h$(GW_{id}\|U_{id}\left\|w\right)=Sk_{ug}⊕Sk_{ug}^{\#}$
  $\;\;\;\;APM_{j}=APM_{j}^{\#}⊕h$$\left(GW_{id}\|U_{id}\|w\right)$
  h$\left(Sk_{gs}\right)=APM_{j}^{{}^{\prime }}⊕APM_{j}$
  $\;\;\;\;SK=h\left(Sk_{gs}\right)⊕SK^{{}^{\prime }}$
  Thus, attacker A has successfully seized the session key SK.

This result shows that Adavoudi-Jolfaei et al.’s scheme does not satisfy the requirement of key security.

### 5.2. Analysis of Sharma and Kalra’s Scheme

#### 5.2.1. Design Error in Sharma and Kalra’s Scheme

There is a fatal error in Sharma and Kalra’s paper. The design of their scheme is wrong. During the authentication phase of their scheme, the session keys computed by SC and GWN are not identical. If the session key is not the same, when authentication is finished and the message is transmitted, there is a problem, because encryption is not properly performed. That is, mutual authentication would be processed incorrectly. We describe this problem in detail.

$SK_{p}$ is the session key that the practitioner generates. $SK_{GWN}$ is the session key that GWN calculates. When sending and receiving messages later, this session key is encrypted.

$\;\;\;\;\;SK_{p}=h\left(N⊕M^{{}^{\prime }}\right)$

$\;\;\;\;\;\;\;\;\;\;=h(N⊕(V_{4}⊕h(Sk_{gs}^{{}^{\prime }}\|T_{1}\|T_{2})))$

$\;\;\;\;\;\;\;\;\;\;=h(N⊕h(Sk_{gs}\|T_{1}\|T_{2})⊕M⊕h(Sk_{gs}^{{}^{\prime }}\|T_{1}\|T_{2}))$

$\;\;\;\;\;\;\;\;\;\;=h(N⊕M⊕h(Sk_{gs}\|T_{1}\|T_{2})⊕h(Sk_{gs}^{{}^{\prime }}\|T_{1}\|T_{2}))$

$\;\;\;\;\;SK_{GWN}=h\left(N^{{}^{\prime }}⊕M\right)$

$\;\;\;\;\;\;\;\;\;\;=h(\left(V_{2}⊕h\left(b^{{}^{\prime }}\|T_{1}\right)\right)⊕M)$

$\;\;\;\;\;\;\;\;\;\;=h(N⊕h\left(b\|T_{1}\right)⊕h\left(b^{{}^{\prime }}\|T_{1}\right)⊕M)$

$\;\;\;\;\;\;\;\;\;\;=h(N⊕h\left(h\left(ID_{p}\|K\right)\|T_{1}\right)⊕h\left(h\left(ID_{p}^{{}^{\prime }}\|K\right)\|T_{1}\right)⊕M)$

$\;\;\;\;\;\;\;\;\;\;=h(N⊕M⊕h\left(h\left(ID_{p}\|K\right)\|T_{1}\right)⊕h\left(h\left(V_{1}⊕h\left(h\left(K\right)\|T_{1}\right)\|K\right)\|T_{1}\right))$

$\;\;\;\;\;\;\;\;\;\;=h(N⊕M⊕h\left(h\left(ID_{p}\|K\right)\|T_{1}\right)⊕h(h\left(ID_{p}⊕h\left(h\left(K\right)\|T_{1}\right)⊕h\left(h\left(K\right)\|T_{1}\right)\|K\right)$

$\;\;\;\;\;\;\;\;\;\;=h(N⊕M⊕h\left(h\left(ID_{p}\|K\right)\|T_{1}\right)⊕h\left(h\left(ID_{p}\|K\right)\|T_{1}\right))$

=h(N⊕M)

In this phase, $Sk_{gs}=h\left(SN_{id}\|K\right)$, but $Sk_{gs}=h\left(SN_{id}\right)$. Therefore, the session keys computed by SC and GWN are not the same. Therefore, the authentication phase should be changed as follows.

1. If *P* logs in successfully, SC computes $b=d⊕h(Mask\left(PW_{p}\right)\|a)$, $h\left(K\right)=c⊕h(Mask\left(PW_{p}\right)\|b)$, $V_{1}=ID_{p}⊕h\left(h\left(K\right)\|T_{1}\right)$. SC selects a random nonce *N* and calculates $V_{2}=N⊕h\left(b\|T_{1}\right)$, $V_{3}=h(V_{1}\|V_{2}\left\|N\right\|T_{1})$. Finally, SC posts the message $M_{1}=\{V_{1}$, $V_{2}$, $V_{3}$, $T_{1}$, $SN_{id}\}$ to GWN.
2. GWN checks the timestamp $|T_{1}-T_{c}|<\Delta T$. If it is in range, GWN computes $MSN_{id}=SN_{id}⊕h\left(h\left(K\right)\|T_{2}\right)$ and chooses a random nonce *M*. GWN continues to calculate $V_{4}=h(Sk_{gs}\|T_{1}\left\|T_{2}\right)⊕M$ and $V_{5}=h(SN_{id}\|V_{4}\|T_{1}\|T_{2}\left\|M\right)$. Finally, GWN sends the message $M_{2}=\{V_{1}$, $V_{2}$, $V_{3}$, $V_{4}$, $V_{5}$, $T_{1}$, $T_{2}$, $MSN_{id}\}$ to the sensor node SN.
3. SN checks the validity of $|T_{2}-T_{c}|<\Delta T$. If it is valid, SN continues the operation $SN_{id}^{{}^{\prime }}=MSN_{id}⊕h\left(h\left(K\right)\|T_{2}\right)$ and checks that $SN_{id}\overset{?}{=}SN_{id}^{{}^{\prime }}$. SN keep calculating as follows:
  $\;\;\;\;M^{{}^{\prime }}=V_{4}⊕h(Sk_{gs}\|T_{1}\left\|T_{2}\right)$
  $\;\;\;\;V_{5}^{{}^{\prime }}=h(SN_{id}\|V_{4}\|T_{1}\|T_{2}\left\|M^{{}^{\prime }}\right)$
  $\;\;\;\;ID_{p}^{{}^{\prime }}=V_{1}⊕h\left(h\left(K\right)\|T_{1}\right)$
  $\;\;\;\;b^{{}^{\prime }}=h\left(ID_{p}^{{}^{\prime }}\|K\right)$
  $\;\;\;\;N^{{}^{\prime }}=V_{2}⊕h\left(b^{{}^{\prime }}\|T_{1}\right)$
  $\;\;\;\;V_{3}^{{}^{\prime }}=h(V_{1}\|V_{2}\|N^{{}^{\prime }}\left\|T_{1}\right)$
  $\;\;\;\;V_{6}=M^{{}^{\prime }}⊕h\left(b^{{}^{\prime }}\|T_{3}\right)$
  $\;\;\;\;V_{7}=N^{{}^{\prime }}⊕h\left(Sk_{gs}\|T_{3}\right)$
  $\;\;\;\;V_{8}=h(V_{6}\|b^{{}^{\prime }}\left\|T_{3}\right)$
  $\;\;\;\;V_{9}=h(V_{7}\|Sk_{gs}\left\|T_{3}\right)$
  Finally, SN sends the message $M_{3}=\{V_{6}$, $V_{7}$, $V_{8}$, $V_{9}$, $T_{3}\}$ to GWN.
4. GWN checks the timestamp $|T_{3}-T_{c}|<\Delta T$ and if it is valid, computes as follows:
  $\;\;\;\;V_{9}^{{}^{\prime }}=h(V_{7}\|Sk_{gs}\left\|T_{3}\right)$
  $\;\;\;\;N^{{}^{\prime }}=V_{7}⊕h\left(Sk_{gs}\|T_{3}\right)$
  $\;\;\;\;SK_{GWN}=h\left(N^{{}^{\prime }}⊕M\right)$
  $\;\;\;\;V_{10}=h(SK_{GWN}\|V_{6}\|V_{8}\|T_{3}\left\|T_{4}\right)$
  At the end of the computation, GWN sends the message $M_{4}=\{V_{6}$, $V_{8}$, $V_{10}$, $T_{3}$, $T_{4}\}$ to *P*.
5. *P* checks the timestamp $|T_{4}-T_{c}|<\Delta T$. If it is in range, it computes $V_{8}^{{}^{\prime }}=h(V_{6}\left\|b\right\|T_{3})$. It also computes $M^{{}^{\prime }}=V_{6}⊕h\left(b\|T_{3}\right)$, $SK_{p}=h\left(N⊕M^{{}^{\prime }}\right)$ and $V_{10}^{{}^{\prime }}=h(SK_{p}\|V_{6}\|V_{8}\|T_{3}\left\|T_{4}\right)$.

In addition to pointing out the correctness problem in Sharma and Kalra’s scheme, as discussed in Section 5.2.1, we demonstrate several attack methods that are fatal to the scheme. We describe the methods in detail as follows:

#### 5.2.2. Password Guessing Attack

In the registration phase, if Attacker A masquerades as GWN, then he/she can easily obtain *P*’s $PW_{p}$. $PW_{p}$ is hashed only once in $Mask(PW_{p})$ with *R*. We assume that *R* can be extracted from the smart device, and the attacker knows the target user’s identity. Moreover, then, Attacker A knows the practitioner *P*’s $ID_{p}$, and he/she can extract the information in SC using reverse engineering or a side-channel attack.

1. Attacker A extracts *a* and *R* from *P*’s smart device SC.
2. A compares *a* and $h(h\left(PW_{p}\|R\right)\|ID_{p})$, so that he/she can guess the password in a brute force attack.

Easily guessing a password implies knowing the practitioner’s identity and likely, passwords, and having access to his/her smart device, so it is virtually the same as a practitioner. This process can also be used to pretend to be a medical professional and directly engage with the patient’s healthcare-related information.

In order to make passwords difficult to guess, the authentication process should involve more robust data encryption.

#### 5.2.3. Stealing the Session Key

We found that the session key $SK_{GWN}$ can be extracted if attacker A used attacks Section 5.2.2. The details are as follows.

1. A computes $Mask\left(PW_{p}\right)=h\left(PW_{p}\|R\right)$, $b=d⊕h(Mask\left(PW_{p}\right)\|a)$.
2. A steals the message $M_{1}$ and extracts $V_{2}$, $T_{1}$. Then, he/she calculates $N=V_{2}⊕h\left(b\|T_{1}\right)$.
3. A also steals $V_{6}$ and $T_{3}$ in $M_{3}$ and computes $M=V_{6}⊕h\left(b\|T_{3}\right)$.
4. Finally, he/she finds the session key $SK_{GWN}=h\left(N⊕M\right)$.

A now has the session key to use in future messages. This session key allows attackers to check the messages. This process is a serious breach of confidentiality.

#### 5.2.4. Sensor Impersonation Attack

A sensor impersonation attack is also possible. Since GWN only assesses the validity of the timestamp to check the sensor separately, the attacker can impersonate the sensor by sending just the timestamp. This attack generates meaningless data and wastes time.

## 6. Proposed Scheme

To address the problems of Sharma and Kalra’s scheme and Adavoudi-Jolfaei et al.’s scheme, we propose a three-factor-based authentication scheme. We specifically introduce a new factor that is based on the practitioner’s biometrics data. In addition, our scheme contains a procedure to validate both GWN and the sensor. The flow of the entire scheme is shown in the Algorithm 1.
**Algorithm 1** Proposed Scheme (Overall Algorithm Flow) 1:$Mask(PW_{p})$, $ID_{p}\leftarrow$ RegistrationP($ID_{p},PW_{p},B_{p}$)      ▹ registration phase 2:*a*, *c*, d← RegistrationGWN($Mask(PW_{p})$, $ID_{p}$) 3:*a*, *c*, *d*, *R*, $P_{bp}$, Gen, Rep, h← RegistrationP2(*a*, *c*, *d*)          ▹ stores in SC 4:**if** LoginP($ID_{p},PW_{p},B_{p}^{\prime }$) **then**                   ▹ login phase 5:    $V_{1}$, $V_{2}$, $V_{3}$, $T_{1}$, $SN_{id}\leftarrow$ AuthenticationP($ID_{p},PW_{p},B_{p}^{\prime }$)  ▹ authentication phase 6:    $V_{1}$, $V_{2}$, $V_{3}$, $V_{4}$, $V_{5}$, $T_{1}$, $T_{2}$, $MSN_{id}\leftarrow$ AuthenticationGWN($V_{1}$, $V_{2}$, $V_{3}$, $T_{1}$, $SN_{id}$) 7:    $V_{6}$, $V_{7}$, $V_{10}$, $T_{3}\leftarrow$ AuthenticationSN($V_{1}$, $V_{2}$, $V_{3}$, $V_{4}$, $V_{5}$, $T_{1}$, $T_{2}$, $MSN_{id}$) 8:    $V_{6}$, $V_{8}$, $V_{11}$, $T_{3}$, $T_{4}\leftarrow$ AuthenticationGWN2($V_{6}$, $V_{7}$, $V_{10}$, $T_{3}$) 9:    Result ← AuthenticationP2($V_{6}$, $V_{8}$, $V_{11}$, $T_{3}$, $T_{4}$)10:    **if** Result **then**11:        **return** Authenticationsuccesses.12:    **end if**13:**end if**

### 6.1. Setup and Registration Phase

#### 6.1.1. Setup Phase

In the setup phase, the gateway node GWN gets its secret key *K* from the registration center. The center also computes $Sk_{gs}=h\left(SN_{id}\|K\right)$ and gives it to the sensor node SN. $Sk_{gs}$ and *K* are stored in GWN and SN.

#### 6.1.2. Registration Phase

The practitioner creates his/her identity and password, and then registers them via the gateway node to receive a smart device. This is summarized in the Algorithm 2. The detailed procedure is as follows:

1. Practitioner *P* chooses his/her identity $ID_{p}$, password $PW_{p}$ and imprints $B_{p}$ over a device for biometrics collection, and calculates a biometric information of the practitioner *P* like Gen ($B_{p})=(R_{p}$, $P_{bp})$. He/she generates random number *R* and computes the masked password $Mask\left(PW_{p}\right)=h\left(h\left(PW_{p}\|R\right)\|R_{p}\right)$. Finally, he/she sends the registration message $\{Mask\left(PW_{p}\right)$, $ID_{p}\}$ to the gateway node GWN.
2. After the gateway node GWN obtains the message from the practitioner, it computes as follows:
  $\;\;\;\;a=h(Mask\left(PW_{p}\right)\|ID_{p})$
  $\;\;\;\;b=h(ID_{p}\|K)$, $c=h\left(K\right)⊕h(Mask\left(PW_{p}\right)\|b)$
  $\;\;\;\;d=b⊕h(Mask\left(PW_{p}\right)\|a)$
  After calculation, GWN sends the smart device SC={a, *c*, d} to the practitioner *P*.
3. *P* stores {a, *c*, *d*, *R*, $P_{bp}$, Gen, Rep, h} in the smart device SC.

**Algorithm 2** Proposed Scheme (Registration Phase) 1:**procedure**RegistrationP($ID_{p},PW_{p},B_{p}$)         ▹ P’s registration phase 2:    $(R_{p}$, $P_{bp})\leftarrow Gen$ ($B_{p})$ 3:    $Mask\left(PW_{p}\right)\leftarrow h\left(h\left(PW_{p}\|R\right)\|R_{p}\right)$ 4:    **return** $Mask(PW_{p})$, $ID_{p}$                 ▹ message to GWN 5:**end procedure** 6:**procedure**RegistrationP2(*a*, *c*, *d*)            ▹ P’s registration phase 2 7:    **return** *a*, *c*, *d*, *R*, $P_{bp}$, Gen, Rep, *h*                ▹ stores in SC 8:**end procedure** 9:**procedure**RegistrationGWN($Mask\left(PW_{p}\right),ID_{p}$)  ▹ GWN’s registration phase10:    $a\leftarrow h(Mask\left(PW_{p}\right)\|ID_{p})$11:    $b\leftarrow h(ID_{p}\|K)$12:    $c\leftarrow h\left(K\right)⊕h(Mask\left(PW_{p}\right)\|b)$13:    $d\leftarrow b⊕h(Mask\left(PW_{p}\right)\|a)$14:    **return** *a*, *c*, *d*                         ▹ message to P15:**end procedure**

### 6.2. Login and Authentication Phase

#### 6.2.1. Login Phase

When the practitioner enters his/her identity and password, the smart device checks whether the practitioner is an authorized party. This phase is summarized in the Algorithm 3. The detailed procedure is as follows:

1. *P* inputs his/her identity $ID_{p}$, password $PW_{p}$ and biometric information $B_{p}^{\prime }$ in his/her smart device.
2. The smart device SC executes the biometric information $R_{p}$ = Rep ($B_{p}^{\prime }$, $P_{bp}$). SC computes masked password and the value *a* as follows:
  $\;\;\;\;Mask\left(PW_{p}\right)=h\left(h\left(PW_{p}\|R\right)\|R_{p}\right)$
  $\;\;\;\;a^{{}^{\prime }}=h\left(Mask\left(PW_{p}\right)\|ID_{p}\right)$
  If *a* and $a^{{}^{\prime }}$ are not the same, *P* fails to login.

**Algorithm 3** Proposed Scheme (Login Phase) 1:**procedure**LoginP($ID_{p},PW_{p},B_{p}^{\prime }$)  ▹ P’s login phase 2:    $R_{p}\leftarrow Rep$ ($B_{p}^{\prime }$, $P_{bp}$) 3:    $Mask\left(PW_{p}\right)\leftarrow h\left(h\left(PW_{p}\|R\right)\|R_{p}\right)$ 4:    $a^{{}^{\prime }}\leftarrow h\left(Mask\left(PW_{p}\right)\|ID_{p}\right)$ 5:    **if** *a* = $a^{{}^{\prime }}$ **then** 6:        **return** True          ▹ login successes 7:    **else** 8:        **return** False             ▹ login fails 9:    **end if**10:**end procedure**

#### 6.2.2. Authentication Phase

The practitioner’s login information and the sensor node execute a mutual authentication process. This phase is summarized in Algorithms 4–6. The procedure is as follows:

1. If *P* logs in successfully, SC selects a random nonce *N* and computes as follows:
  $\;\;\;\;b=d⊕h(Mask\left(PW_{p}\right)\|a)$
  $\;\;\;\;h\left(K\right)=c⊕h(Mask\left(PW_{p}\right)\|b)$
  $\;\;\;\;V_{1}=ID_{p}⊕h\left(h\left(K\right)\|T_{1}\right)$
  $\;\;\;\;V_{2}=N⊕h\left(b\|T_{1}\right)$
  $\;\;\;\;V_{3}=h(V_{1}\|V_{2}\left\|N\right\|T_{1})$
  Finally, SC sends the message $M_{1}=\{V_{1}$, $V_{2}$, $V_{3}$, $T_{1}$, $SN_{id}\}$ to GWN.
2. GWN checks the timestamp $|T_{1}-T_{c}|<\Delta T$. If it is in range, GWN computes as follows:
  $\;\;\;\;ID^{{}^{\prime }}=V_{1}⊕h\left(h\left(K\right)\|T_{1}\right)$
  $\;\;\;\;V_{3}^{{}^{\prime }}=h(V_{1}^{{}^{\prime }}\|V_{2}\|\left(V_{2}⊕h\left(h\left(ID^{{}^{\prime }}\|K\right)\|T_{1}\right)\right)\left\|T_{1}\right)$
  GWN also checks $V_{3}=V_{3}^{{}^{\prime }}$. If it is valid, GWN chooses a random nonce *M* and computes as follows:
  $\;\;\;\;MSN_{id}=SN_{id}⊕h\left(h\left(K\right)\|T_{2}\right)$
  $\;\;\;\;V_{4}=h(Sk_{gs}\|T_{1}\left\|T_{2}\right)⊕M$
  $\;\;\;\;V_{5}=h(SN_{id}\|V_{4}\|T_{1}\|T_{2}\left\|M\right)$
  Finally, GWN sends the message $M_{2}=\{V_{1}$, $V_{2}$, $V_{3}$, $V_{4}$, $V_{5}$, $T_{1}$, $T_{2}$, $MSN_{id}\}$ to the sensor node SN.
3. SN checks the validity of $|T_{2}-T_{c}|<\Delta T$. If it is valid, SN continues the operation $SN_{id}^{{}^{\prime }}=MSN_{id}⊕h\left(h\left(K\right)\|T_{2}\right)$ and checks $SN_{id}^{{}^{\prime }}\overset{?}{=}SN_{id}$. SN computes as follows:
  $\;\;\;\;M^{{}^{\prime }}=V_{4}⊕h(Sk_{gs}\|T_{1}\left\|T_{2}\right)$
  $\;\;\;\;V_{5}^{{}^{\prime }}=h(SN_{id}\|V_{4}\|T_{1}\|T_{2}\left\|M^{{}^{\prime }}\right)$
  It also checks the validity of $V_{5}^{{}^{\prime }}\overset{?}{=}V_{5}$. If it is valid, SN computes:
  $\;\;\;\;ID^{{}^{\prime }}=V_{1}⊕h\left(h\left(K\right)\|T_{1}\right)$
  $\;\;\;\;b^{{}^{\prime }}=h\left(ID^{{}^{\prime }}\|K\right)$, $N^{{}^{\prime }}=V_{2}⊕h\left(b^{{}^{\prime }}\|T_{1}\right)$
  $\;\;\;\;V_{6}=M^{{}^{\prime }}⊕h\left(b^{{}^{\prime }}\|T_{3}\right)$, $V_{7}=N^{{}^{\prime }}⊕h\left(Sk_{gs}\|T_{3}\right)$
  $\;\;\;\;V_{8}=h(V_{6}\|b^{{}^{\prime }}\left\|T_{3}\right)$
  $\;\;\;\;V_{9}=h(V_{7}\|Sk_{gs}\left\|T_{3}\right)$
  $\;\;\;\;V_{10}=h(V_{8}\|V_{9}\left\|T_{3}\right)$
  Finally, SN sends the message $M_{3}=\{V_{6}$, $V_{7}$, $V_{10}$, $T_{3}\}$ to GWN.
4. GWN checks the timestamp $|T_{3}-T_{c}|<\Delta T$ and if it is valid, GWN computes:
  $\;\;\;\;V_{8}^{{}^{\prime }}=h(V_{6}\left\|b\right\|T_{3})$
  $\;\;\;\;V_{9}^{{}^{\prime }}=h(V_{7}\|Sk_{gs}\left\|T_{3}\right)$
  $\;\;\;\;V_{10}^{{}^{\prime }}=h(V_{8}^{{}^{\prime }}\|V_{9}^{{}^{\prime }}\left\|T_{3}\right)$ and checks $V_{10}\overset{?}{=}V_{10}^{{}^{\prime }}$. If it is also valid, GWN computes:
  $\;\;\;\;N^{{}^{\prime }}=V_{7}⊕h\left(Sk_{gs}\|T_{3}\right)$
  $\;\;\;\;SK_{GWN}=h\left(N^{{}^{\prime }}⊕M\right)$
  $\;\;\;\;V_{11}=h(SK_{GWN}\|V_{6}\|V_{8}\|T_{3}\left\|T_{4}\right)$
  At the end of the computation, GWN sends the message $M_{4}=\{V_{6}$, $V_{8}$, $V_{11}$, $T_{3}$, $T_{4}\}$ to *P*.
5. *P* checks the timestamp $|T_{4}-T_{c}|<\Delta T$. If it is in range, *P* computes $V_{8}^{{}^{\prime }}=h(V_{6}\left\|b\right\|T_{3})$ and checks $V_{8}\overset{?}{=}V_{8}^{{}^{\prime }}$. *P* also computes $M^{{}^{\prime }}=V_{6}⊕h\left(b\|T_{3}\right)$, $SK_{p}=h\left(N⊕M^{{}^{\prime }}\right)$ and checks $V_{11}^{{}^{\prime }}=h(SK_{p}\|V_{6}\|V_{8}\|T_{3}\left\|T_{4}\right)\overset{?}{=}V_{11}$.

**Algorithm 4** Proposed Scheme (P’s Authentication Phase) 1:**procedure**AuthenticationP($ID_{p},PW_{p},B_{p}^{\prime }$)    ▹ P’s authentication phase 2:    $R_{p}\leftarrow Rep$ ($B_{p}^{\prime }$, $P_{bp}$) 3:    $Mask\left(PW_{p}\right)\leftarrow h\left(h\left(PW_{p}\|R\right)\|R_{p}\right)$ 4:    $a^{{}^{\prime }}\leftarrow h\left(Mask\left(PW_{p}\right)\|ID_{p}\right)$ 5:    $b\leftarrow d⊕h(Mask\left(PW_{p}\right)\|a)$ 6:    $h\left(K\right)\leftarrow c⊕h(Mask\left(PW_{p}\right)\|b)$ 7:    $V_{1}\leftarrow ID_{p}⊕h\left(h\left(K\right)\|T_{1}\right)$ 8:    $V_{2}\leftarrow N⊕h\left(b\|T_{1}\right)$ 9:    $V_{3}\leftarrow h(V_{1}\|V_{2}\left\|N\right\|T_{1})$10:    **return** $V_{1}$, $V_{2}$, $V_{3}$, $T_{1}$, $SN_{id}$              ▹ message to GWN11:**end procedure**12:**procedure**AuthenticationP2($V_{6}$, $V_{8}$, $V_{11}$, $T_{3}$, $T_{4}$)  ▹ P’s authentication phase 213:    **if** $|T_{4}-T_{c}|>=\Delta T$ **then**14:        **return** False                   ▹ authentication fails15:    **end if**16:    $V_{8}^{{}^{\prime }}\leftarrow h(V_{6}\left\|b\right\|T_{3})$17:    **if** $V_{8}!=V_{8}^{{}^{\prime }}$ **then**18:        **return** False                   ▹ authentication fails19:    **end if**20:    $M^{{}^{\prime }}\leftarrow V_{6}⊕h\left(b\|T_{3}\right)$21:    $SK_{p}\leftarrow h\left(N⊕M^{{}^{\prime }}\right)$22:    $V_{11}^{{}^{\prime }}\leftarrow h(SK_{p}\|V_{6}\|V_{8}\|T_{3}\left\|T_{4}\right)$23:    **if** $V_{11}^{{}^{\prime }}!=V_{11}$ **then**24:        **return** False                   ▹ authentication fails25:    **end if**26:    **return** True                  ▹ authentication successes27:**end procedure**
**Algorithm 5** Proposed Scheme (GWN’s Authentication Phase) 1:**procedure**AuthenticationGWN($V_{1}$, $V_{2}$, $V_{3}$, $T_{1}$, $SN_{id}$) ▹ GWN’s authentication phase 2:    **if** $|T_{1}-T_{c}|>=\Delta T$ **then** 3:        **return** False                       ▹ authentication fails 4:    **end if** 5:    $ID^{{}^{\prime }}\leftarrow V_{1}⊕h\left(h\left(K\right)\|T_{1}\right)$ 6:    $V_{3}^{{}^{\prime }}\leftarrow h(V_{1}^{{}^{\prime }}\|V_{2}\|\left(V_{2}⊕h\left(h\left(ID^{{}^{\prime }}\|K\right)\|T_{1}\right)\right)\left\|T_{1}\right)$ 7:    **if** $V_{3}!=V_{3}^{{}^{\prime }}$ **then** 8:        **return** False                       ▹ authentication fails 9:    **end if**10:    $MSN_{id}\leftarrow SN_{id}⊕h\left(h\left(K\right)\|T_{2}\right)$11:    $V_{4}\leftarrow h(Sk_{gs}\|T_{1}\left\|T_{2}\right)⊕M$12:    $V_{5}\leftarrow h(SN_{id}\|V_{4}\|T_{1}\|T_{2}\left\|M\right)$13:    **return** $V_{1}$, $V_{2}$, $V_{3}$, $V_{4}$, $V_{5}$, $T_{1}$, $T_{2}$, $MSN_{id}$             ▹ message to SN14:**end procedure**15:**procedure**AuthenticationGWN2($V_{6}$, $V_{7}$, $V_{10}$, $T_{3}$)  ▹ GWN’s authentication phase 216:    **if** $|T_{3}-T_{c}|>=\Delta T$ **then**17:        **return** False                      ▹ authentication fails18:    **end if**19:    $V_{8}^{{}^{\prime }}\leftarrow h(V_{6}\left\|b\right\|T_{3})$20:    $V_{9}^{{}^{\prime }}\leftarrow h(V_{7}\|Sk_{gs}\left\|T_{3}\right)$21:    $V_{10}^{{}^{\prime }}\leftarrow h(V_{8}^{{}^{\prime }}\|V_{9}^{{}^{\prime }}\left\|T_{3}\right)$22:    **if** $V_{10}!=V_{10}^{{}^{\prime }}$ **then**23:        **return** False                      ▹ authentication fails24:    **end if**25:    $N^{{}^{\prime }}\leftarrow V_{7}⊕h\left(Sk_{gs}\|T_{3}\right)$26:    $SK_{GWN}\leftarrow h\left(N^{{}^{\prime }}⊕M\right)$27:    $V_{11}\leftarrow h(SK_{GWN}\|V_{6}\|V_{8}\|T_{3}\left\|T_{4}\right)$28:    **return** $V_{6}$, $V_{8}$, $V_{11}$, $T_{3}$, $T_{4}$                    ▹ message to P29:**end procedure**
**Algorithm 6** Proposed Scheme (SN’s Authentication Phase) 1:**procedure**AuthenticationSN($V_{1}$, $V_{2}$, $V_{3}$, $V_{4}$, $V_{5}$, $T_{1}$, $T_{2}$, $MSN_{id}$)    ▹ SN’s authentication phase 2:    **if** $|T_{2}-T_{c}|>=\Delta T$ **then** 3:        **return** False                            ▹ authentication fails 4:    **end if** 5:    $SN_{id}^{{}^{\prime }}\leftarrow MSN_{id}⊕h\left(h\left(K\right)\|T_{2}\right)$ 6:    **if** $SN_{id}^{{}^{\prime }}!=SN_{id}$ **then** 7:        **return** False                            ▹ authentication fails 8:    **end if** 9:    $M^{{}^{\prime }}\leftarrow V_{4}⊕h(Sk_{gs}\|T_{1}\left\|T_{2}\right)$10:    $V_{5}^{{}^{\prime }}\leftarrow h(SN_{id}\|V_{4}\|T_{1}\|T_{2}\left\|M^{{}^{\prime }}\right)$11:    **if** $V_{5}^{{}^{\prime }}!=V_{5}$ **then**12:        **return** False                            ▹ authentication fails13:    **end if**14:    $ID^{{}^{\prime }}\leftarrow V_{1}⊕h\left(h\left(K\right)\|T_{1}\right)$15:    $b^{{}^{\prime }}\leftarrow h\left(ID^{{}^{\prime }}\|K\right)$, $N^{{}^{\prime }}=V_{2}⊕h\left(b^{{}^{\prime }}\|T_{1}\right)$16:    $V_{6}\leftarrow M^{{}^{\prime }}⊕h\left(b^{{}^{\prime }}\|T_{3}\right)$17:    $V_{7}\leftarrow N^{{}^{\prime }}⊕h\left(Sk_{gs}\|T_{3}\right)$18:    $V_{8}\leftarrow h(V_{6}\|b^{{}^{\prime }}\left\|T_{3}\right)$19:    $V_{9}\leftarrow h(V_{7}\|Sk_{gs}\left\|T_{3}\right)$20:    $V_{10}\leftarrow h(V_{8}\|V_{9}\left\|T_{3}\right)$21:    **return** $V_{6}$, $V_{7}$, $V_{10}$, $T_{3}$                          ▹ message to GWN22:**end procedure**

### 6.3. Password Change Phase

The practitioner can change his/her password. The procedure is as follows:

1. *P* inputs his/her identity $ID_{p}$, password $PW_{p}$ and biometric information $B_{p}^{\prime }$ in his/her smart device.
2. The smart device SC executes Rep ($B_{p}^{\prime }$, $P_{bp}$) = $R_{p}$. SC computes $Mask\left(PW_{p}\right)=h\left(h\left(PW_{p}\|R\right)\|R_{p}\right)$, $a^{*}=h\left(Mask\left(PW_{p}\right)\|ID_{p}\right)$. SC checks $a^{*}\overset{?}{=}a$. If so, it computes $b=d⊕h(Mask\left(PW_{p}\right)\|a)$, $h\left(K\right)=h(Mask\left(PW_{p}\right)\|b)⊕c$. Finally, SC sends the message {Enternewpassword} to *P*.
3. *P* inputs his/her new password $PW_{p}^{new}$ to the smart device SC.
4. SC computes $Mask{\left(PW_{p}\right)}^{{}^{\prime }}=h\left(R\|PW_{p}^{new}\right)$, $a^{{}^{\prime }}=h\left(Mask{\left(PW_{p}\right)}^{{}^{\prime }}\|ID_{p}\right)$, $d^{{}^{\prime }}=b⊕h\left(Mask{\left(PW_{p}\right)}^{{}^{\prime }}\|a^{{}^{\prime }}\right)$, $c^{{}^{\prime }}=h\left(K\right)⊕h\left(Mask{\left(PW_{p}\right)}^{{}^{\prime }}\|b^{{}^{\prime }}\right)$. Finally, SC replaces {a, *c*, d} with $\{a^{{}^{\prime }}$, $c^{{}^{\prime }}$, $d^{{}^{\prime }}\}$.

## 7. Security Analysis

In this section, we analyze the security of the proposed scheme in two ways: formal security analysis and informal security analysis. We use a formal protocol verification tool called ProVerif in Section 7.1, to demonstrate the security of our scheme. We also provide a theoretical security analysis of the protocol in Section 7.2. Through this verification, we have demonstrated how safe our scheme can be in reality.

### 7.1. Formal Security Analysis

We use ProVerif to analyze the security and correctness of our scheme; ProVerif is widely used to verify security protocols [7,30,31]. ProVerif is a software tool that formally verifies the security of cryptographic protocols. We define basic cryptographic primitives such as hash function, encryption, digital signature and bit-commitment.

We used three channels: a registration channel (cha), a practitioner–gateway node channel (chb) and a gateway node–sensor node channel (chc). The variables, constants, secret key, functions and events are defined in Table A1.

The “Registration” and “Login and Authentication” phases for practitioners are shown in Table A2. The “Registration” and “Authentication” phases for gateway nodes are shown in Table A3. The “Authentication” phase for sensor nodes is shown in Table A4. Table 2 and Table 3 show a query and the corresponding query results.

When we run the query in Table 2, we obtain the following result:

1. RESULT inj-event(EVENTA) ==> inj-event(EVENTB) is true.
2. RESULT inj-event(EVENTA) ==> inj-event(EVENTB) is false.
3. RESULT not attacker(QUERY) is true.
4. RESULT not attacker(QUERY) is false.

“RESULT inj-event (EVENTA) == > inj-event (EVENTB) is true.” means that EVENTA to EVENTB has been authenticated. On the other hand, “RESULT inj-event (EVENTA) == > inj-event (EVENTB) is false.” means that the authentication from EVENTA to EVENTB was not successful. “RESULT not attacker (QUERY) is true.” means that an attacker cannot get a free name QUERY, and “RESULT not attacker (QUERY) is false.” means that an attacker can trace a QUERY [32].

The results of the query in Table 2 are shown in Table 3. In that case, the authentication process is correctly performed and the attacker cannot obtain IDp.

### 7.2. Informal Security Analysis

We present a theoretical analysis of our scheme. We then briefly explain the results of the analysis.

#### 7.2.1. Privileged Insider Attack

In the registration step, the practitioner sends his/her plaintext ID and $Mask\left(PW_{p}\right)=h\left(h\left(PW_{p}\|R\right)\|R_{p}\right)$ to the gateway node. Since the ID is used in conjunction with secret information *K* without being exposed to the outside, there is no way for an insider to know the practitioner’s personal information. Therefore, it is secure against a privileged insider attack.

#### 7.2.2. Outsider Attack

The SC only contains the information {a, *c*, *d*, *R*, $P_{bp}$, Gen, Rep, h}. We cannot infer practitioner *P* in this case.

#### 7.2.3. Offline ID Guessing Attack

The practitioner’s identity is not moved directly in the login and authentication phase after registration. When the sensor checks the practitioner’s ID, the information $V_{1}$, *K*, and $T_{1}$ is required, all of which cannot be obtained through SC.

#### 7.2.4. Online ID Guessing Attack

The practitioner’s identity can only be exposed as $V_{1}$, *K*, and $T_{1}$, as mentioned in the offline ID guessing attack. At this time, $V_{1}$ and $T_{1}$ can be seized through a message sent to the GWN by the practitioner in the authentication phase, but the secret GWN key *K* cannot be found.

#### 7.2.5. Session Key Disclosure Attack

As shown in Section 5.2.1, the session key consists of h(N⊕M). *N* is a value generated by SC and $N=V_{2}⊕h\left(b\|T_{1}\right)$, and *M* is a value generated by GWN and expressed as $M=V_{4}⊕h(X_{GWN}\|T_{1}\left\|T_{2}\right)$. Because the attacker is not the practitioner or GWN, he/she cannot know the session key because $X_{GWN}$ including *b* and Mask(PW) including *K* cannot be known.

#### 7.2.6. Practitioner Impersonation Attack

GWN verifies the practitioner in equation $V_{3}^{{}^{\prime }}=h(V_{1}^{{}^{\prime }}\|V_{2}\|V_{2}⊕h\left(h\left(ID^{{}^{\prime }}\|K\right)\|T_{1}\right)\|T_{1})\overset{?}{=}V_{3}=h(ID_{p}⊕h\left(h\left(K\right)\|T_{1}\right)\|N⊕h\left(b\|T_{1}\right)\left\|N\right\|T_{1})$. If an attacker pretends to be an authorized practitioner, then he/she needs to know b=d⊕h(Mask(PW)‖a), h(K)=c⊕h(Mask(PW)‖b). However, the attacker cannot obtain Mask(PW). Therefore, the attacker cannot impersonate the practitioner *P*.

#### 7.2.7. Sensor Impersonation Attack

GWN verifies a sensor with the Equation $V_{10}=h(V_{8}\|V_{9}\left\|T_{3}\right)$ and checks $V_{10}\overset{?}{=}V_{10}^{{}^{\prime }}$ that $V_{8}=h(V_{6}\left\|b\right\|T_{3})$, $V_{9}=h(V_{7}\|Sk_{gs}\left\|T_{3}\right)$. This means that a sensor can only prove that it is a sensor if it knows $Sk_{gs}$ and *b*.

## 8. Performance Analysis of the Proposed Scheme

**Figure 2:**
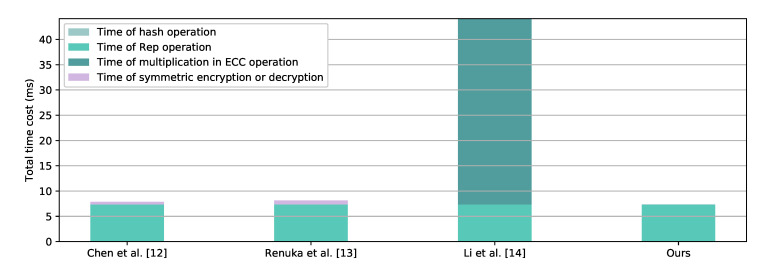
Execution Time of Schemes.

The four symbols necessary for comparison are as follows. $T_{Rep}$ is the time to check for a match when recognizing the user’s (or practitioner’s) biometric $B_{p}$. $T_{h}$ is the time it takes to hash. $T_{m}$ is the time of the multiplicative operation used in ECC. The time taken for symmetric encryption or decryption is denoted by $T_{s}$. These are listed in Table 4. We compared our scheme with the following three schemes of Chen et al. [12], Renuka et al. [13] and Li et al. [14]. The four symbols necessary for comparison are as follows, and depicted as a graph in Figure 2.

Table 5 summarizes the total time cost for each scheme ([12,13,14]). The Y-axis shown in Figure 2 is the total time cost in microseconds (ms). This is calculated based on the times shown in Table 4. Table 6 shows the computer hardware and software used to calculate the algorithm’s runtime. Li et al.’s scheme [14] uses elliptic curve cryptography, however it has large time costs because of its slow encryption and decryption.

The methods used in Chen et al.’s [12] and Renuka et al.’s [13] scheme have lower costs than Li et al.’s [14]. They used symmetric encryption and decryption, but had a significant difference in cost compared to ours.

## 9. Conclusions

Recently, several lightweight two-factor-based authentication protocols, such as Sharma and Kalra’s protocol [6] and Adavoudi-Jolfaei et al.’s protocol [11], were proposed for IoT applications. Those protocols have some benefits in efficiency because they are designed with light and straightforward operations, such as hash function and XOR without complicated cryptographic operations. However, we found that those protocols have several security weaknesses.

To address these problems, we use three factors: the practitioner’s identity, a password, and biometric information. We propose a lightweight three-factor user authentication scheme to fix several issues in Sharma and Kalra’s protocol [6] and Adavoudi-Jolfaei et al.’s protocol [11]. We provide the security verification of the proposed scheme using ProVerif. Furthermore, we show that our proposed method outperforms other proposed symmetric-based or elliptic curve-based methods.

We are motivated to fix the problems of existing protocols and proposed a more efficient and secure authentication scheme. Our scheme is designed only with the hash function and XOR, providing fast and secure authentication. The proposed protocol can be used for various IoT applications such as medical devices.

Our proposed scheme can be implemented with simple operations, but introduces 11 new parameters. Furthermore, while our scheme is lighter than the symmetric-based or elliptic curve-based methods, it is not significantly lighter than the only used XOR and hash method. Therefore, in future work, we aim to develop a simpler authentication scheme with fewer variables. We also aim to develop a lightweight 3-factor authentication scheme with fewer xor operations and hash functions. Our efforts will contribute to creating a faster and more secure authentication scheme.

## Figures and Tables

**Table 1:** Notations.

Notations	Description
*U*	The user
*P*	The practitioner who is a medical professional
GWN	Gateway node
SN	Sensor node
$U_{id}$	*U*’s identity
$U_{psw}$	*U*’s password
$U_{b}$	*U*’s biometric information
$AU_{id}$	*U*’s disposable identity
$SU_{id}$	*U*’s shadow identity
APM	Set of access rights mask for *U*
*G*	Group identity set of *U*
$B_{p}$	Biometric information of the practitioner *P*
$ID_{p}$	Identity of the practitioner *P*
$PW_{p}$	Password of the practitioner *P*
$Mask(PW_{p})$	Masked password of the user *P*
$GWN_{id}$	GWN’s identity
*w*	GWN’s private key
$KEM_{ug}$	The secret emergency key between *U* and GWN
$Sk_{ug}$	The secret key between *U* and GWN
$SN_{id}$	SN’s identity
$Sk_{gs}$	The secret key between GWN and SN
SK	The session key between *U* and SN
SC	Smart card or smart device
DB	Database
$Ts_{ug}$	Timestamp sequence
$Mask(PW_{p})$	Masked password of the user *P*
*K*	Secret key of GWN
h(·)	One-way hash function
$T_{x}$	The $x^{th}$ timestamp
$T_{c}$	Current timestamp
Gen	A probabilistic generation function
Rep	A deterministic reproduction function
ΔT	Maximum transmission delay
⊕	XOR operation
‖	Concatenation operation

**Table 2:** Query.

(*—-queries—-*)
query attacker(IDp).
query id:bitstring; inj-event(endP(id)) ==> inj-event(beginP(id)).
query id:bitstring; inj-event(endGWN(id)) ==> inj-event(beginGWN(id)).
query id:bitstring; inj-event(endS(id)) ==> inj-event(beginS(id)).
process
((!P)|(!GWN)|(!S))

**Table 3:** Query Results.

RESULT inj-event(endS(id)) ==> inj-event(beginS(id)) is true.
RESULT inj-event(endGWN(id_21256)) ==> inj-event(beginGWN(id_21256)) is true.
RESULT inj-event(endP(id_41657)) ==> inj-event(beginP(id_41657)) is true.
RESULT not attacker(IDp[]) is true.

**Table 4:** Notations of Time Symbol.

Symbol	Meaning	Time (ms)
$T_{Rep}$	time of Rep	7.3529 [33]
$T_{h}$	time of hash operation	0.0004 [34]
$T_{m}$	time of multiplication in ECC	7.3529 [34]
$T_{s}$	time of symmetric encryption or decryption	0.1303 [34]

**Table 5:** Comparison of Computation Costs.

	Chen et al. [12]	Renuka et al. [13]	Li et al. [14]	Ours
User(Practitioner) *P*	9$T_{h}$ + 1$T_{Rep}$ + 1$T_{s}$	5$T_{h}$ + 1$T_{Rep}$ + 2$T_{s}$	8$T_{h}$ + 1$T_{Rep}$ + 2$T_{m}$	13$T_{h}$ + 1$T_{Rep}$
GWN	3$T_{h}$ + 2$T_{s}$	2$T_{h}$+ 2$T_{s}$	7$T_{h}$ + 1$T_{m}$	14$T_{h}$
Sensor node $S_{j}$	4$T_{h}$ + $T_{s}$	3$T_{h}$ + 2$T_{s}$	4$T_{h}$ + 2$T_{m}$	12$T_{h}$
Total time cost	16$T_{h}$+ 4$T_{s}$+ 1$T_{Rep}$	10$T_{h}$+ 6$T_{s}$ + 1$T_{Rep}$	19$T_{h}$ + 5$T_{m}$ + 1$T_{Rep}$	39$T_{h}$ + 1$T_{Rep}$
(ms)	=7.8805	=8.1387	=44.125	=7.3685

**Table 6:** Hardware and Software Condition.

	Specification
CPU	Intel (R) Core(TM) 2T6570 2.1GHz
Memory	4G
OS	Win7 32-bit
Software	Visual C++ 2008

## Appendix A. ProVerif Code

**Table A1:** Define Values and Functions.

(*—-channels—-*)
free cha:channel [private].
free chb:channel.
free chc:channel.
(*—-constants—-*)
free Ru:bitstring [private].
free IDp:bitstring [private].
free IDg:bitstring [private].
free IDs:bitstring.
free PWu:bitstring [private].
(*—-secret key—-*)
free K:bitstring [private].
(*—-functions—-*)
fun concat(bitstring, bitstring): bitstring.
fun xor(bitstring, bitstring): bitstring.
fun h(bitstring): bitstring.
equation forall a:bitstring, b:bitstring; xor(xor(a, b), b) = a.
(*—-events—-*)
event beginP(bitstring).
event endP(bitstring).
event beginGWN(bitstring).
event endGWN(bitstring).
event beginS(bitstring).
event endS(bitstring).

**Table A2:** Practitioner Scheme.

(*—-P process—-*)
let P =
new R:bitstring;
let MPW = h(concat(h(concat(PWp, R)), Rp)) in
out(cha,(IDp, MPW));
in(cha,(Xa:bitstring, Xc:bitstring, Xd:bitstring));
event beginP(IDp);
new T1:bitstring;
new N:bitstring;
let ppb = xor(Xd, h(concat(MPW, Xa))) in
let hK = xor(Xc, h(concat(MPW, ppb))) in
let V1 = xor(IDp, h(concat(hK, T1))) in
let V2 = xor(N, h(concat(ppb, T1))) in
let V3 = h(concat(concat(V1, V2), concat(N, T1))) in
out(chb, (V1, V2, V3, T1, N));
in(chb, (XXV6:bitstring, XXV8:bitstring, XV11:bitstring, XXT3:bitstring, XT4:bitstring));
let pV8 = h(concat(concat(XXV6, ppb), XXT3)) in
let pM = xor(XXV6, h(concat(ppb, XXT3))) in
let SKp = h(xor(N, pM)) in
let pV11 = h(concat(concat(SKp, XXV6), concat(concat(XXV8, XXT3),XT4))) in
event endP(IDp).

**Table A3:** Gateway Node Scheme.

(*—-GWN process—-*)
let GWN =
in(cha,(XIDp:bitstring, XMPW:bitstring));
let a = h(concat(XMPW, XIDp)) in
let b = h(concat(XIDp, K)) in
let c = xor(h(K), h(concat(XMPW, b))) in
let d = xor(b, h(concat(XMPW, a))) in
out(cha, (a, c, d));
in(chb, (XV1:bitstring, XV2:bitstring, XV3:bitstring, XT1:bitstring, XN:bitstring));
event beginGWN(IDg);
new T2:bitstring;
new M:bitstring;
let pIDp = xor(XV1, h(concat(h(K), XT1))) in
let pV3 = h(concat(concat(XV1, XV2), concat(XN, XT1))) in
if pV3 = XV3 then
let MID = xor(IDs, h(concat(h(K), T2))) in
let X = h(concat(IDs, K)) in
let V4 = xor(M, h(concat(concat(X, XT1), T2))) in
let V5 = h(concat(concat(IDs, V4), concat(concat(XT1, T2), M))) in
out(chc, (XV1, XV2, XV3, V4, V5, XT1, T2, MID));
in(chc, (XV6:bitstring, XV7:bitstring, XV10:bitstring, XT3:bitstring));
new T4:bitstring;
new M:bitstring;
let pV8 = h(concat(XV6, concat(b, XT3))) in
let pV9 = h(concat(XV7, concat(X, XT3))) in
let pV10 = h(concat(pV8, concat(pV9, XT3))) in
if pV10 = XV10 then
let pN = xor(XV7, h(concat(X, XT3))) in
let SKg = h(xor(pN, M)) in
let V11 = h(concat(SKg, concat(concat(XV6, pV8), concat(XT3, T4)))) in
out(chb, (XV6, pV8, V11, XT3, T4));
event endGWN(IDg).

**Table A4:** Sensor Scheme.

(*—-S process—-*)
let S =
in(chc, (XXV1:bitstring, XXV2:bitstring, XXV3:bitstring, XV4:bitstring, XV5:bitstring,
XXT1:bitstring, XT2:bitstring, XMID:bitstring));
event beginS(IDs);
new T3:bitstring;
let XX = h(concat(IDs, K)) in
let pM = xor(XV4, h(concat(concat(XX, XXT1), XT2))) in
let pV5 = h(concat(concat(IDs, XV4), concat(concat(XXT1, XT2), pM))) in
if pV5 = XV5 then
let pb = h(concat(IDp, K)) in
let ppN = xor(XXV2, h(concat(pb, XXT1))) in
let V6 = xor(pM, h(concat(pb, T3))) in
let V7 = xor(ppN, h(concat(XX, T3))) in
let V8 = h(concat(concat(V6, pb), T3)) in
let V9 = h(concat(concat(V7, XX), T3)) in
let V10 = h(concat(concat(V8, V9), T3)) in
out(chc, (V6, V7, V10, T3));
event endS(IDs).

## References

[1] Gregg M. Trends in Remote Patient Monitoring 2019. Spyglass Consulting Group. http://www.spyglass-consulting.com/wp_RPM_2019.html.

[2] Hu Y.H., Tompkins W.J., Urrusti J.L., Afonso V.X. (1993). Applications of artificial neural networks for ECG signal detection and classification. J. Electrocardiol..

[3] Yeh Y.C., Wang W.J. (2008). QRS complexes detection for ECG signal: The Difference Operation Method. Comput. Methods Programs Biomed..

[4] Van Ess D.W. (2006). ECG Signal Detection Device. US Patent.

[5] Chung W.Y., Lee Y.D., Jung S.J. A wireless sensor network compatible wearable u-healthcare monitoring system using integrated ECG, accelerometer and SpO_2_. Proceedings of the 2008 30th Annual International Conference of the IEEE Engineering in Medicine and Biology Society.

[6] Sharma G., Kalra S. (2019). A Lightweight User Authentication Scheme for Cloud-IoT Based Healthcare Services. Iran. J. Sci. Technol. Trans. Electr. Eng..

[7] Ryu J., Lee H., Kim H., Won D. (2018). Secure and efficient three-factor protocol for wireless sensor networks. Sensors.

[8] Rathore H., Al-Ali A., Mohamed A., Du X., Guizani M. DTW based authentication for wireless medical device security. Proceedings of the 2018 14th International Wireless Communications & Mobile Computing Conference (IWCMC).

[9] Ali R., Pal A.K., Kumari S., Sangaiah A.K., Li X., Wu F. (2018). An enhanced three factor based authentication protocol using wireless medical sensor networks for healthcare monitoring. J. Ambient. Intell. Humaniz. Comput..

[10] Choi Y., Lee D., Kim J., Jung J., Nam J., Won D. (2014). Security enhanced user authentication protocol for wireless sensor networks using elliptic curves cryptography. Sensors.

[11] Adavoudi-Jolfaei A., Maede A.T., Aghili S.F. (2019). Lightweight and anonymous three-factor authentication and access control scheme for real-time applications in wireless sensor networks. Peer-to-Peer Netw. Appl..

[12] Chen Y., Ge Y., Wang Y., Zeng Z. (2019). An improved three-factor user authentication and key agreement scheme for wireless medical sensor networks. IEEE Access.

[13] Renuka K., Kumar S., Kumari S., Chen C.M. (2019). Cryptanalysis and improvement of a privacy-preserving three-factor authentication protocol for wireless sensor networks. Sensors.

[14] Li X., Niu J., Bhuiyan M.Z.A., Wu F., Karuppiah M., Kumari S. (2017). A robust ECC-based provable secure authentication protocol with privacy preserving for industrial internet of things. IEEE Trans. Ind. Inform..

[15] Hu F., Jiang M., Wagner M., Dong D.C. (2007). Privacy-preserving telecardiology sensor networks: Toward a low-cost portable wireless hardware/software codesign. IEEE Trans. Inf. Technol. Biomed..

[16] Malasri K., Wang L. (2009). Design and implementation of a securewireless mote-based medical sensor network. Sensors.

[17] Kumar P., Lee S.G., Lee H.J. (2012). E-SAP: Efficient-strong authentication protocol for healthcare applications using wireless medical sensor networks. Sensors.

[18] Khan M.K., Kumari S. (2014). An improved user authentication protocol for healthcare services via wireless medical sensor networks. Int. J. Distrib. Sens. Netw..

[19] Li X., Niu J., Kumari S., Liao J., Liang W., Khan M.K. (2016). A new authentication protocol for healthcare applications using wireless medical sensor networks with user anonymity. Secur. Commun. Netw..

[20] Wu F., Xu L., Kumari S., Li X. (2017). An improved and anonymous two-factor authentication protocol for health-care applications with wireless medical sensor networks. Multimed. Syst..

[21] Hossain M.S., Muhammad G. (2015). Cloud-assisted speech and face recognition framework for health monitoring. Mob. Netw. Appl..

[22] Wazid M., Das A.K., Shetty S., Rodrigues J.J.P.C., Park Y. (2019). LDAKM-EIoT: Lightweight device authentication and key management mechanism for edge-based IoT deployment. Sensors.

[23] Tanveer M., Zahid A.H., Ahmad M., Baz A., Alhakami H. (2020). LAKE-IoD: Lightweight Authenticated Key Exchange Protocol for the Internet of Drone Environment. IEEE Access.

[24] Gope P., Tzonelih H. (2016). A realistic lightweight anonymous authentication protocol for securing real-time application data access in wireless sensor networks. IEEE Trans. Ind. Electron..

[25] Katz J., Menezes A.J., Van Oorschot P.C., Vanstone S.A. (1996). Handbook of Applied Cryptography.

[26] Dodis Y., Katz J., Reyzin L., Smith A. (2006). Robust fuzzy extractors and authenticated key agreement from close secrets. Annual International Cryptology Conference.

[27] Dodis Y., Reyzin L., Smith A. (2004). Fuzzy extractors: How to generate strong keys from biometrics and other noisy data. International Conference on the Theory and Applications of Cryptographic Techniques.

[28] Dolev D., Yao A. (1983). On the security of public key protocols. IEEE Trans. Inf. Theory.

[29] Moon J., Lee D., Lee Y., Won D. (2017). Improving biometric-based authentication schemes with smart card revocation/reissue for wireless sensor networks. Sensors.

[30] Lee H., Lee D., Moon J., Jung J., Kang D., Kim H., Won D. (2018). An improved anonymous authentication scheme for roaming in ubiquitous networks. PLoS ONE.

[31] Wu F., Li X., Sangaiah A.K., Xu L., Kumari S., Wu L., Shen J. (2018). A lightweight and robust two-factor authentication scheme for personalized healthcare systems using wireless medical sensor networks. Future Gener. Comput. Syst..

[32] Blanchet B., Smyth B., Cheval V., Sylvestre M. (2018). ProVerif 2.00: Automatic Cryptographic Protocol Verifier, User Manual and Tutorial. https://prosecco.gforge.inria.fr/personal/bblanche/proverif/manual.pdf.

[33] Das A.K. (2016). A secure and robust temporal credential-based three-factor user authentication scheme for wireless sensor networks. Peer-to-Peer Netw. Appl..

[34] Xu L., Wu F. (2015). Cryptanalysis and improvement of a user authentication scheme preserving uniqueness and anonymity for connected health care. J. Med. Syst..

